# Event-level association between alcohol use and unprotected sex during last sex: evidence from population-based surveys in sub-Saharan Africa

**DOI:** 10.1186/1471-2458-13-583

**Published:** 2013-06-15

**Authors:** Susan M Kiene, SV Subramanian

**Affiliations:** 1 Department of Community Medicine and Health Care, University of Connecticut School of Medicine, Farmington, CT, USA; 2 Department of Global Health and Population, Harvard School of Public Health, Boston, MA, USA; 3 Department of Society, Human Development, and Health, Harvard School of Public Health, Boston, MA, USA

**Keywords:** Africa, Alcohol, HIV

## Abstract

**Background:**

HIV and risky alcohol use are intertwined public health issues in sub-Saharan Africa. Research supports the association between alcohol and unprotected sex, but there is limited data using event-level analysis to examine this relationship.

**Methods:**

Using data from Demographic Health Surveys and AIDS Information Surveys collected in 8 sub-Saharan African countries (Kenya, Lesotho, Mozambique, Rwanda, Swaziland, Tanzania, Zambia, and Zimbabwe) drunkenness (reporting male partner or both male and female partner being drunk during last sexual intercourse) at last sex was tested as a predictor of unprotected last sex among the male (n = 24,512) and female (n = 28,229) participants. Partner type, HIV test results, and the other variables were evaluated as effect modifiers of this relationship.

**Results:**

Drunkenness at last sex had a negative effect on the likelihood of condom use among men (AOR 0.84, 95% CI 0.72-0.99) and a marginally significant effect among women (AOR 0.87, 95% CI 0.59-1.02) in Southern Africa. However, for men in Southern Africa, this effect was primarily observed with steady partners. Contrary to predictions, in both Southern and Eastern Africa, for men, drunkenness during sex with casual partners increased the odds of condom use.

**Conclusions:**

These data indicate a need to implement HIV prevention efforts that consider the role of alcohol use in precipitating unprotected sex and how it varies based upon partner type.

## Background

The relationship between alcohol consumption and HIV has spawned a growing body of literature, with mounting support suggesting not only that the two are related [1-5] but that they should be treated as intertwined public health issues. The “fuel to the fire” nature of the relationship between alcohol consumption and HIV is evidenced throughout sub-Saharan Africa, which has 67% of the world’s HIV infections, [6] as well as countries with high rates of risky drinking patterns, such as drinking until intoxicated and heavy episodic drinking [7]. Risky drinking is a predictor of sexual risk behavior in sub-Saharan Africa [5,8] and is common throughout the region. The World Health Organization (WHO) estimates heavy episodic drinking, commonly defined as consuming five or more drinks in one sitting, to occur at least once weekly among 25% of drinkers in Africa, which is double the worldwide average of 11.5% [7].

Understanding the relationship between alcohol consumption and HIV is a crucial step in addressing the HIV epidemic. While the biological consequences of alcohol consumption, such as a weakening of the immune system [9] and an increase in an HIV positive individual’s viral load, [10] have been noted as important avenues of transmission, the social consequences may be even more important. That is, alcohol’s facilitation of unprotected sex may increase the potential for HIV-infection. Several mechanisms have been theorized to explain this association, including the physiological effects of alcohol on decision making [11,12], expectations regarding alcohol intake and condom use [13,14], as well as certain personality types being more likely to engage in risky behaviors [15-17].

The vast majority of evidence supporting an association between alcohol consumption and unprotected sex is based on either *global associations,* which correlate self-reports of overall frequency of alcohol consumption or *situational-overlap associations*, which analyze the frequency of drinking during sex with self-reported frequency of unprotected sex [1,18]. These methodologies do not permit the researcher to determine if drinking during a specific sexual intercourse event is specifically associated with failure to use a condom during that sexual event. This limitation can be addressed by using *event-level analysis*, which requires pairing alcohol consumption during a specific instance of sexual intercourse and whether or not a condom was used.

Earlier literature reviews of studies based in the US and other developed nations found no association at the event-level, [19,20] however, more recent meta-analyses that included both developed and developing nations have found support for an event-level association between alcohol use prior to or during sex with risk of HIV [1,21] and unprotected sex [18]. Furthermore, if data is collected on which partner is consuming alcohol, as well as on the quantity of alcohol consumed or level of intoxication, using event-level analyses allows the researcher to determine gender differences and if alcohol exerts effects only at certain levels of intoxication. For example, Kiene et al. [22] found that drinking alcohol before sex by the female, the male, or by both partners, increased the proportion and number of subsequent unprotected sex events, but only when the quantity of alcohol consumed was at a moderate or higher risk drinking level. Despite strong support for the use of event-level analysis, little research exists that looks at alcohol use and risky sexual behavior at the event-level and even less that have been conducted in sub-Saharan Africa. Of the studies that meet this description to date [22-24], a significant association between alcohol use prior to or during sex and unprotected sex has been demonstrated.

The present analysis extends the relatively limited literature, none of which derives from population-based data, on the event-level association between alcohol consumption during sex and unprotected sex by testing this association across sub-Saharan African countries using data from Demographic and Health Survey (DHS) and AIDS Indicator Survey (AIS) population-based surveys. We hypothesize that drunkenness at last sex will be associated with lower odds of condom use, especially when sex is with a casual partner.

## Methods

The current study analyzes DHS and AIS data from 8 eastern and southern sub-Saharan African countries collected between 2005 and 2009. DHS are nationally-representative cross-sectional population-level household surveys of between 5,000 and 30,000 households conducted in developing countries by host governments with support from IFC International and funded by the United States Agency for International Development and participating countries [25]. AIS use the same methods as DHS but focus on a smaller range of health topics (e.g., HIV/AIDS). DHS/AIS data are widely used for research, policymaking, and program development in population health.

Countries’ data were included in the analysis if the DHS/AIS survey met the following criteria: located in Eastern or Southern sub-Saharan Africa (regions which have the highest HIV prevalence in Africa); included HIV-testing linked to survey data, information on condom use at last sex, and self and partner drunkenness during last sexual intercourse. Ethiopia (2005) was excluded because it had very low rates (under 1%) of respondents reporting that they and/or their partner consumed alcohol at last sex. Countries included in the analysis were: Kenya (2008–2009) [26], Lesotho (2009) [27], Mozambique (2009) [28], Rwanda (2005) [29], Swaziland (2006–2007) [30], Tanzania (2007–2008) [31], Zambia (2007) [32], and Zimbabwe (2005–2006) [33]. Lesotho was excluded from the female sample because its DHS did not collect data about drunkenness at last sex from female respondents. We used DHS survey data for all countries, with the exception of Mozambique for which we used AIDS Indicator Survey (AIS) data, and Tanzania for which we used the HIV/AIDS and Malaria Indicator Survey (AIS/MIS) data.

The DHS and AIS data collection procedures were approved by the ORC Macro/Macro International (Calverton, Maryland) Institutional Review Board as well as by the ethics committee in each country responsible for approving human subjects research studies. In the original study, oral informed consent was obtained from respondents by interviewers. The secondary analysis of this data for the present study was determined by The University of Connecticut Institutional Review Board to not constitute human subjects research because the data was from an anonymous public use data set with no identifiable information on the participants and therefore did not require further ethics review.

Surveys used for the current analysis include data from women age 15–49 and men age 15–59. Exceptions include Kenya and Zimbabwe where men 15–54 were sampled and Mozambique, Swaziland, and Tanzania where men 15–49 were sampled. Further details about sampling and data collection procedures are available in the individual country reports.

### Measures

DHS/AIS survey items of interest in the present study are questions about the last sexual intercourse event: self and/or partner drunkenness (“Were you or your partner drunk at that time?:” respondent only, partner only, both respondent and partner, neither), condom use (“The last time you had sexual intercourse with this person, was a condom used?:” yes vs. no), relationship to sex partner (“What was your relationship to this person with whom you had sexual intercourse?:” spouse, live-in partner, boy/girlfriend, casual, friend, commercial sex worker/client, other) and the HIV test results (measured through a blood test; the DHS and AIS measures do not capture one’s knowledge of his or her HIV positive status). For men, drunkenness at last sex was operationalized as either the respondent reporting being drunk or the respondent reporting that both he and his partner were drunk (questionnaire item responses: respondent only or both respondent and partner). For women, it was operationalized as either the respondent reporting that her partner was drunk or that both she and her partner were drunk (questionnaire item responses: partner only or both respondent and partner). We operationalized drinking in this way because of the very low frequency of events where the female but not the male partner was drunk (see Table 1). We included only instances where respondents rated that they or their partner were “drunk” and excluded instances where they reported consuming alcohol but not being drunk because the latter occurred at low frequency (1.9% and 2.8% in the female and male samples, respectively). Steady partners were considered to be spouses, live-in partners, non-cohabitating boy/girlfriends, or fiancées. Casual partners were considered to be casual acquaintances, “other friends,” (for men) commercial sex workers, and (for women) paying clients.

**Table 1:** Sample socio-demographics and descriptive statistics (men: n = 24,512, women: n = 28,229)

	**East Africa**		**Southern Africa**	
	**(Kenya, Mozambique, Rwanda, Tanzania)**		**(Lesotho, Swaziland, Zambia, Zimbabwe)**	
	**Men**	**Women**	**Men**	**Women**
N	12,772	16,032	11,740	12,197
Average age (SD)	33.77 (10.74)	30.85 (9.34)	32.05 (10.40)	29.44 (8.61)
Marital Status				
Married/cohabitating	76.35%	81.94%	64.28%	76.49%
Not married	23.65%	18.06%	35.72%	23.51%
Education				
High school or >	24.13%	16.12%	52.10%	48.75%
Primary	62.13%	58.75%	40.46%	42.99%
No schooling	13.74%	25.13%	7.43%	8.26%
Urban residence	30.21%	29.21%	33.71%	33.67%
Rural residence	69.79%	70.79%	66.29%	66.33%
Wealth index				
Poorest	15.44%	16.62%	18.79%	18.57%
Poorer	17.99%	17.22%	19.00%	19.18%
Middle	19.06%	18.47%	19.42%	19.80%
Richer	21.04%	22.11%	23.07%	22.22%
Richest	26.46%	25.57%	20.70%	20.23%
HIV prevalence	6.38%	8.22%	17.17%	23.74%
Casual partner at last sex	5.36%	1.24%	4.05%	0.69%
Condom use last sex	18.46%	8.74%	25.32%	16.55%
Respondent (men)/ partner (women) or both drunk during last sex	6.07%	8.77%	10.96%	11.08%
Respondent only drunk during last sex	4.85%	0.62%	9.88%	0.65%
Partner only drunk during last sex	0.62%	7.71%	0.33%	10.40%
Both drunk during last sex	1.22%	1.06%	1.07%	0.68%
Sex with non-spousal, non-cohabitating partner(s) (prior year)	35.24%	17.86%	36.97%	21.77%
Know condoms prevent HIV	80.55%	73.50%	82.96%	82.11%

Potential covariates evaluated in the analyses include questions about: sex with a non-marital, non-cohabitating partner in the prior 12 months and knowledge of condom use as an effective HIV prevention method, as well as sociodemographic characteristics: country, age, urban vs. rural residence, marital status, education, and income. Income is operationalized in DHS surveys as a “wealth index” which is represented on a 5-point scale from poorest to richest with each country’s sample being divided into quintiles. Further details about the construction of this variable can be found in the individual DHS country reports.

### Data analysis approach

For frequencies and descriptive statistics reported in Table 1, cases were weighted based on pooled weights derived from the proportion (with valid HIV test results) of the total country mid-year population during the survey year. We present results by sub-Saharan Africa region (Eastern: Kenya, Mozambique, Rwanda, and Tanzania; and Southern: Lesotho, Swaziland, Zambia, and Zimbabwe) instead of by country because, especially in countries with lower HIV prevalence, the cell sizes for comparing frequencies of condom use based on drunkenness and partner type are too small to permit valid comparisons. Mozambique was included in the eastern region instead of the southern because its HIV prevalence and prevalence of drunkenness at last sex was more similar to the countries in the eastern region than the southern region.

We conducted the analyses using SPSS 18.0 [34]. Drunkenness at last sex was tested as a predictor of using a condom during last sex using binomial generalized linear regression models with a logit link. Partner type at last sex and HIV test results were evaluated as effect modifiers of this relationship. Covariates described above were tested as predictors of condom use at last sex and as effect modifiers of the association between drunkenness and condom use.

## Results

The male analytic sample comprised 24,512 (59.49% of the entire sample) and the female sample comprised 28,229 (49.91% of the entire sample) with available data on the exposure, outcome and covariates. Socio-demographic data for each region by gender is presented in detail in Table 1. The average age of participants across both regions ranged from 29–34 years of age. The majority of men and women from both East and Southern Africa reported being married/cohabitating (range 64-82%) and residing in a rural area (range 66-71%). About half of men (52%) and approximately 49% of women in Southern Africa attained a high school education or higher, compared to 24% of men and 16% of women in East Africa. In East Africa the largest proportion had completed primary school (62% of men, 59% of women). The percentage reporting no schooling was 7 to 8% among women and men in Southern Africa and 14-25% among women and men in East Africa. Additional descriptive statistics by region and gender are also presented in Table 1, including HIV prevalence, condom use at last sex, drunkenness at last sex, and HIV risk and preventive factors. HIV prevalence was higher in Southern Africa among men and women (men: 17.17%, women: 23.74%) compared to East Africa (men: 6.38%, women: 8.22%). In both regions, women had higher HIV prevalence than their male counterparts. Condom use at last sex was also higher in Southern Africa (men: 25.32%, women: 15.55%) compared to East Africa (men: 18.46%, women: 8.74%), as was male partner or both male and female drunkenness at last sex (Southern: men: 10.96%, women: 11.08%; East: men: 6.07%, women: 8.77%). See Table 1 for more details.

### Demographic and behavioral correlates of using a condom at last sex

Results of the multivariate regression testing potential predictors of condom use at last sex is presented in detail in Table 2 and are summarized below. Odds ratios are adjusted for the inclusion of all variables in the regression model and 95% confidence intervals are presented in the table. In all regions for both men and women the following two variables were associated with a reduced likelihood of using a condom at last sex: increasing age and being married/cohabitating, whereas the following two demographic variables were associated with an increased likelihood of using a condom at last sex: higher education and higher economic status. In Eastern Africa residing in an urban area vs. a rural area was associated with higher odds of using a condom at last sex for both men and women but there were no differences based on residence type in Southern Africa. We also found differences between countries within each region. In the male sample in Southern Africa, the odds of condom use at last sex were greater in both Lesotho (AOR 2.23, 95% CI 1.96-2.55) and Swaziland (AOR 2.00, 95% CI 1.75-2.25) compared to Zambia (reference) but there were no differences between Zimbabwe and Zambia. Among women, with Zambia as the reference group, the odds of condom use at last sex were greater in Swaziland (AOR 2.27, 95% CI 1.99-2.61) but less in Zimbabwe (AOR 0.56, 95% CI 0.48-0.66). In the male sample in Eastern Africa, condom use at last sex was more likely in Kenya (AOR 1.92, 95% CI 1.62-2.27) and Tanzania (AOR 1.61, 95% CI 1.38-1.88) compared to Mozambique but there were no statistically significant differences between Rwanda and Mozambique. Among women, with Mozambique as the reference group, condom use at last sex was more likely in Tanzania (AOR 1.46, 95% CI 11.24-1.72) but less likely in Rwanda (AOR 0.61, 95% CI 0.47-0.80) and there were no differences between Kenya and Mozambique.

**Table 2:** Adjusted associations of using a condom at last sex for men and women by sub-Saharan African Region

	**East Africa**		**Southern Africa**	
	**(Kenya, Mozambique, Rwanda, Tanzania)**		**(Lesotho**^**‡**^**, Swaziland, Zambia, Zimbabwe)**	
	**Men**	**Women**	**Men**	**Women**
	**n = 12,772**	**n = 16,032**	**n = 11,740**	**n = 12,197**
	**AOR (95% CI)**	**AOR (95% CI)**	**AOR (95% CI)**	**AOR (95% CI)**
Age, per year	**0.97 (0.96-0.98)**	**0.96 (0.95-0.97)**	**0.98 (0.98-0.99)**	**0.99 (0.98-0.99)**
Marital status				
Married/cohabitating vs. unmarried (ref)	**0.36 (0.30-0.43)**	**0.37 (0.29-0.46)**	**0.28 (0.24-0.32)**	**0.41 (0.32-0.53)**
Education (ref: No schooling)				
High school or >	**2.25 (1.74-2.91)**	**2.63 (2.06-3.37)**	**1.92 (1.56-2.37)**	**2.04 (1.58-2.65)**
Primary	**1.33 (1.05-1.69)**	**1.53 (1.24-1.89)**	**1.29 (1.05-1.58)**	**1.44 (1.12-1.87)**
Residence				
Urban vs. rural (ref)	**1.37 (1.18-1.59)**	**1.36 (1.16-1.61)**	1.09 (0.96-1.23)	1.04 (0.90-1.20)
Wealth index	**1.20 (1.14-1.27)**	**1.17 (1.10-1.24)**	**1.18 (1.13-1.24)**	**1.25 (1.18-1.32)**
Country				
Kenya (East) / Lesotho^‡^ (South)	**1.92 (1.62-2.27)**	0.99 (0.81-1.22)	**2.23 (1.96-2.55)**	-
Tanzania (East) / Swaziland (South)	**1.61 (1.38-1.88)**	**1.46 (1.24-1.72)**	**2.00 (1.75-2.25)**	**2.27 (1.99-2.61)**
Rwanda (East) / Zimbabwe (South)	0.80 (0.63-1.01)^†^	**0.61 (0.47-0.80)**	1.06 (0.94-1.20)	**0.56 (0.48-0.66)**
Mozambique (East) (ref) / Zambia (South) (ref)	-	-	-	-
Knows condoms prevent HIV				
Yes vs. no (ref)	**1.84 (1.56-2.18)**	**1.71 (1.43-2.05)**	**1.44 (1.26-1.65)**	**1.48 (1.25-1.76)**
Sex with non-spousal, non-cohabitating partner(s) in prior year				
Yes vs. no (ref)	**4.95 (4.15-5.91)**	**4.01 (3.16-5.09)**	**1.89 (1.63-2.17)**	**2.63 (2.03-3.40)**
Male partner or both were drunk during last sex				
Yes vs. no (ref)	0.76 (0.48-1.19)	0.83 (0.51-1.36)	**0.84 (0.72-0.99)**	0.78 (.59-1.02)^†^
Partner type				
Casual vs. steady (ref)	1.16 (0.96-1.40)	1.13 (0.79-1.63)	1.00 (0.80-1.26)	1.22 (0.74-2.01)
HIV test results				
HIV positive vs. HIV negative (ref)	**2.43 (1.97-3.01)**	**2.08 (1.74-2.48)**	**1.53 (1.36-1.72)**	**1.67 (1.48-1.88)**
**Interaction**				
Drunk x partner type	**1.74 (1.01-3.00)**	1.79 (0.67-4.75)	1.57 (0.95-2.54)^†^	0.97 (0.26-3.61)

*Note: AOR* adjusted odds ratio; bold text denotes statistically significant findings at *p < .05,*^†^*p* < .10. Estimates are adjusted for all variables and interactions listed in this table. Country comparisons are within each region. ^‡^ For the female sample there were only 3 countries for comparison within Southern Africa since the Lesotho female data set did not contain data regarding alcohol consumption during last sex.

Across both regions for both men and women the following two behavioral variables were associated with using a condom at last sex: knowing that condoms are effective at preventing HIV infection and reporting having non-spousal, non-cohabitating partners in the prior 12 months; the latter having nearly two to five times the odds of condom use at last sex compared to those not reporting non-spousal, non-cohabitating partners. Those whose HIV test results were HIV positive were between 1.5 and 2.4 times more likely to have used a condom at last sex compared to those whose HIV test results were negative. We did not find differences in the odds of condom use based on partner type, although differences may be reflected in the interaction between drunkenness and partner type as reported below.

### Drunkenness during last sex—condom use association

To test the drunkenness during last sex—condom use association we focused primarily on the male partner drunkenness and compared instances where the male partner was not drunk (reference) vs. instances where the male partner was drunk (including instances where both the male and female partners were drunk in the latter category). As detailed in Table 2, in both the male and female sample in the Southern Africa region, if the male partner (or both partners) was drunk during last sex then they were approximately 1.2 times less likely to use a condom compared to if the male partner was not drunk during last sex (men: AOR 0.84, 95% CI 0.72-0.99; women: AOR 0.78, 95% CI 0.59-1.02). Although, this association was marginally statistically significant (*p* = 0.08) in the female sample.

For men in the Southern and Eastern Africa regions, the effect of drunkenness during last sex on the odds of condom use at last sex varied based on partner type (marginally statistically significant for the Southern Africa region). Specifically, the above interaction in Eastern Africa showed that when the sex event was with a casual partner, drunkenness at last sex was associated with higher odds of condom use (AOR 1.74, 95% CI 1.01-3.00), whereas when the sex event was with a steady partner, drunkenness did not affect the odds of condom use. For Southern Africa, the marginally statistically significant interaction showed that there was a decrease in the odds of condom use at last sex associated with drunkenness for sex with steady partners but for sex with casual partners, drunkenness increased the likelihood of condom use (AOR 1.57, 95% CI 0.95-2.54). Among women, we did not observe a modifying effect of partner type on the drunkenness—condom use at last sex association.

HIV test results did not modify the effect of drunkenness on condom use among men or women. We also found no modifying effects of demographic, country, or HIV behavioral risk and protective factors for men or women on the effect of drunkenness at last sex on odds of condom use.

## Discussion

Event-level analyses determining if drinking occurred during a specific event of sexual intercourse and if a condom was used during that sex event, offer significant advantages in attempting to infer a causal relationship. It allows the researcher to rule out alternative explanations for an alcohol—unprotected sex relationship: that individuals who consume alcohol also have unprotected sex, but they do not drink during sex, or that individuals may drink during sex but drinking does not affect whether or not they use a condom. The current research examined the event-level relationship between drunkenness at last sex and condom use during that sex event among male and female samples from population-based surveys in 8 sub-Saharan African countries.

As expected, drunkenness at last sex had a negative effect on condom use, but not in all situations. In Southern Africa, drunkenness during sex by the male partner or both the male and female partner resulted in lower odds of condom use, although this effect was marginally statistically significant in the female sample. Southern Africa was also the region with the highest prevalence of drunkenness at last sex but the highest overall rates of condom use at last sex. In the male sample in both regions, drunkenness during sex with casual partners increased the likelihood of condom use (marginally significant in the Southern Africa region). This protective effect of alcohol consumption in situations with casual partners is unexpected and contrary to our predictions and some other event level-studies, [22,35-37] but may be explained by a rational risk decision making model—that is, individuals may have expectancies about sex with casual partners—that alcohol will make them less likely to use a condom [14,38] and therefore they prepare in advance to use a condom.

Drunkenness during sex by the female partner, alone or with her partner, was very rare (< 2%) in both regions, therefore, in the female sample, drunkenness during sex primarily reflects men’s drunkenness. Our results from the Southern Africa region provide further support for a prior study’s suggestion that women are put at risk primarily through men’s drinking [3].

Since these data are drawn from population-based surveys the findings can be generalized to countries within the studied regions with similar profiles of HIV prevalence and alcohol consumption before sex.

### Limitations

Due to low HIV prevalence and frequency of drunkenness at last sex, we were not able to analyze the data for each country individually but we did include country as a covariate. Respondents’ knowledge of his or her HIV positive status is not collected in DHS or AIS surveys and therefore we cannot determine if our findings showing higher likelihood of condom use among those who tested HIV positive reflects an increased focus on using condoms among those who know that they are HIV positive. Since drunkenness was ascertained by self-report instead of alcohol quantity measures (e.g., number and type of drinks), this reflects a subjective judgment of intoxication. However, alcohol quantity measures may not necessarily represent an objective level of intoxication either, due to individual variations in alcohol metabolism. Furthermore, the report of partners’ level of drunkenness may be more objective than relying on knowledge of the quantity of alcohol the partner consumed. Capturing alcohol use only at the most recent sexual event may also not be a true reflection of the prevalence of drinking during sex.

Nearly half of the cases in the male and half of the cases in the female sample were excluded from the present analyses because they were missing data on the exposure, outcome, or covariates. This may have introduced sampling bias, especially because of the sizable number of cases that were missing valid HIV test results. Most of these cases are because participants declined or were absent for HIV testing during the original surveys. A significant number of cases were excluded because they did not have data about condom use at last sex which reflects that they had either not had sex within the prior 12 months (in the DHS/AIS surveys the question was skipped if the participant had not had sex within that time frame) or had never had sex. This potential sampling bias limits the generalizability of the findings to individuals who were sexually active within the prior 12 months and would not likely refuse HIV testing.

Furthermore, it is possible that several variables not captured in the dataset could potentially be confounding the relationship between drunkenness and condom use at last sex. With single event-level data, rather than multiple events from each participant, we cannot rule out individual differences (e.g., personality characteristics, behavior patterns) as explanations for observed associations. In a review of the literature, Shuper et al. [39] identified personality characteristics, psychiatric disorders, and situational factors as potential confounders of the relationship between alcohol and risky sex. For example, one recent study found no association between alcohol and unprotected sex at the event-level, but found the personality trait of conscientiousness to be an independent risk factor for both unprotected sex and estimated blood alcohol concentration (eBAC) [16].

Lastly, the low prevalence of drunkenness during last sex (ranging from 6% in Eastern Africa to 11% in Southern Africa in the male sample and from 9% in Eastern Africa to 11% in Southern Africa in the female sample) would seem to limit the importance of our findings from a public health policy perspective. However, the risk of HIV transmission posed by alcohol’s effect on reducing the likelihood of condom use is sizable on a population level in countries with high HIV prevalence. As an illustration, using the present data set which shows that the median frequency of sexual intercourse is every two weeks (assuming frequency of sex is two times the median time since last sex), the percentage of sex acts that involved drunkenness, the results presented in this paper showing an increase in the odds of unprotected sex by approximately 1.2 in Southern Africa due to drunkenness during sex, and considering the population at the time of the survey of the Southern African countries reported on here, nearly 25 million sex events among men in Southern Africa and nearly 28 million sex events among women in Southern Africa each year would be unprotected due to the increased risk posed by alcohol use during sex.

## Conclusion

Despite the limitations, the present study examining the event-level relationship between alcohol consumption and condom use using data from 8 sub-Saharan African countries is the first such study to use population-based data. These data indicate a critical need for HIV prevention efforts for men and women that consider the role of alcohol use in precipitating unprotected sex, especially in Southern Africa and especially for sex with steady partners. Such interventions could teach men who drink alcohol and women whose partners drink alcohol about the impact of alcohol consumption on decision making about both having sex and using a condom and should support men and women in making condom use a normal part of sex, especially when they are drunk.

## Competing interests

The authors declare that they have no competing interests.

## Authors’ contributions

SMK performed the data analyses, wrote the first draft of the manuscript, and along with SVS conceived of the idea and hypothesis. SVS and SMK both contributed to revising the manuscript. All authors read and approved the final manuscript.

## Pre-publication history

The pre-publication history for this paper can be accessed here:

http://www.biomedcentral.com/1471-2458/13/583/prepub
